# Examining cortical tracking of the speech envelope in post-stroke aphasia

**DOI:** 10.3389/fnhum.2023.1122480

**Published:** 2023-09-14

**Authors:** Yina M. Quique, G. Nike Gnanateja, Michael Walsh Dickey, William S. Evans, Bharath Chandrasekaran

**Affiliations:** ^1^Center for Education in Health Sciences, Northwestern University Feinberg School of Medicine, Chicago, IL, United States; ^2^Department of Communication Sciences and Disorders, University of Wisconsin-Madison, Madison, WI, United States; ^3^VA Pittsburgh Healthcare System, Pittsburgh, PA, United States; ^4^Department of Communication Sciences and Disorders, University of Pittsburgh, Pittsburgh, PA, United States; ^5^Roxelyn and Richard Pepper Department of Communication Science and Disorders, School of Communication. Northwestern University, Evanston, IL, United States

**Keywords:** aphasia (language), rhythm, cortical tracking of speech, EEG, Spanish

## Abstract

**Introduction:**

People with aphasia have been shown to benefit from rhythmic elements for language *production* during aphasia rehabilitation. However, it is unknown whether rhythmic processing is associated with such benefits. Cortical tracking of the speech envelope (CTenv) may provide a measure of encoding of speech rhythmic properties and serve as a predictor of candidacy for rhythm-based aphasia interventions.

**Methods:**

Electroencephalography was used to capture electrophysiological responses while Spanish speakers with aphasia (*n* = 9) listened to a continuous speech narrative (audiobook). The Temporal Response Function was used to estimate CTenv in the delta (associated with word- and phrase-level properties), theta (syllable-level properties), and alpha bands (attention-related properties). CTenv estimates were used to predict aphasia severity, performance in rhythmic perception and production tasks, and treatment response in a sentence-level rhythm-based intervention.

**Results:**

CTenv in delta and theta, but not alpha, predicted aphasia severity. Neither CTenv in delta, alpha, or theta bands predicted performance in rhythmic perception or production tasks. Some evidence supported that CTenv in theta could predict sentence-level learning in aphasia, but alpha and delta did not.

**Conclusion:**

CTenv of the syllable-level properties was relatively preserved in individuals with less language impairment. In contrast, higher encoding of word- and phrase-level properties was relatively impaired and was predictive of more severe language impairments. CTenv and treatment response to sentence-level rhythm-based interventions need to be further investigated.

## Introduction

Aphasia is a communication disorder resulting from damage to brain regions that support language. Due to their communication impairments, people with aphasia (PWA) experience social isolation, loss of autonomy, and other consequences that affect their quality of life (Cruice et al., 2006; Hilari, 2011). Intervention approaches leveraging rhythmic aspects of speech and music have been shown to be beneficial in improving language production in PWA (e.g., Stahl et al., 2011, 2013). However, little is known about PWA’s encoding of speech rhythmic properties and their connection to language impairments, rhythmic perception/production, and treatment response to rhythm-based interventions.

The amplitude envelope conveys rhythmic cues that are important for speech perception (Rosen, 1992; Shannon et al., 1995). The neural processing of these cues in continuous speech can be assessed by examining cortical tracking using electroencephalography (EEG). Specifically, cortical tracking of the speech envelope (CTenv) can be used as a proxy to assess the neural processing of rhythmic properties in continuous speech, but has not been previously examined in post-stroke aphasia. CTenv refers specifically to the tracking of the acoustic/linguistic properties of the speech signal by the low-frequency cortical activity in the M/EEG (Ding and Simon, 2014). The cortical activity in the M/EEG reflects activity of populations of neurons (Lakatos et al., 2019; Gnanateja et al., 2022) – essential for processing rhythm cues in speech and music (Peelle et al., 2013; Doelling et al., 2019; Poeppel and Assaneo, 2020; Gnanateja et al., 2022). CTenv could thus open a window into PWA’s encoding of speech rhythmic properties, which could shed light on treatment response to rhythm-based interventions.

CTenv has been examined in the delta, theta, and alpha bands of neural oscillations. The *delta band (~1–4 Hz)* aligns with the word and phrase boundaries in connected speech and prosodic cues, and has been associated with higher-order language processing (Ghitza, 2017; Teoh et al., 2019). The *theta band (∼4–8 Hz)* broadly corresponds to the syllabic rate (5*–*8 Hz) found in multiple languages (Ding et al., 2017) and is key in segmenting auditory information into syllabic chunks (Ghitza, 2013; Kösem and van Wassenhove, 2016; Poeppel and Assaneo, 2020). Activity in this band has been shown to be stronger for intelligible speech (Peelle et al., 2013) than for unintelligible speech (Pefkou et al., 2017); or equal in both types of stimuli (Howard and Poeppel, 2010). The *alpha band (~8–12 Hz)* is associated with attentional processes (Obleser and Weisz, 2012; Wöstmann et al., 2017) and effortful listening (Paul et al., 2021).

CTenv in these bands has been used to investigate neurophysiological mechanisms underlying speech, language, and music processing in neurotypical populations (e.g., Doelling et al., 2014), including in Spanish speakers (e.g., Assaneo et al., 2019; Pérez et al., 2019; Lizarazu et al., 2021). These neurophysiological mechanisms could inform our understanding of how the encoding of rhythmic properties of speech are affected by aphasia and how they may influence treatment response to rhythm-based interventions. Therefore, the current study examines the association between CTenv and aphasia severity, rhythmic perception/production abilities, and treatment response to a sentence-level rhythm-based intervention in post-stroke aphasia.

Recent evidence from primary progressive aphasia (PPA; Dial et al., 2021) demonstrated an increased CTenv in theta but not delta compared to neurotypicals, implying that people with PPA may rely more on acoustic properties during continuous speech processing. To date, this is one of the few studies that investigate CTenv in adult neurogenic language disorders (see De Clercq et al., 2023; Kries et al., 2023). Thus, the association between CTenv and language impairments as measured by aphasia severity is, to our knowledge, yet to be examined.

CTenv may also be associated with rhythmic perception and production measures in individuals with aphasia. Some PWA show difficulties in rhythmic *perception* tasks (Zipse et al., 2014; Sihvonen et al., 2016), such as identifying whether two excerpts with rhythmic variations are the same or different. Some others show more difficulties than controls in rhythmic *production* tasks, such as continuing a rhythmic pattern after its offset – with some PWA showing preserved abilities in synchronizing to a given rhythm (Zipse et al., 2014). Importantly, a better understanding of rhythmic perception and production in aphasia can guide language treatment candidacy, especially for rhythm-based interventions. For example, Curtis et al. (2020) reported that PWA with better rhythmic abilities were more likely to benefit from an additional rhythmic cue (hand tapping) in response to melodic intonation therapy. Similarly, Stefaniak et al. (2021) found a correlation between the perception of rhythmic patterns and picture description fluency in PWA. The association between rhythmic measures and CTenv could help to understand rhythmic performance in aphasia in ways that can support candidacy and treatment response.

Rhythmic elements have been successfully employed in aphasia rehabilitation. For example, Stahl et al. (2011) examined how PWA learned lyrics using three conditions: melodic intoning, rhythmic speech, and a control condition, and reported a comparable degree of benefit between the melodic and rhythmic conditions. A follow-up study by Stahl et al. (2013) showed improved sentence learning using singing and rhythmic therapy. Also, simultaneous production of audiovisual speech, which depends on the synchronization of speech rhythmic properties between a patient and clinician, has resulted in improved sentence learning in PWA (Fridriksson et al., 2012, 2015), which is consistent with previous work on neurotypicals showing that audiovisual speech enhances CTenv and improves speech perception (Chandrasekaran et al., 2009; Crosse et al., 2015). However, it is currently unknown whether the neural encoding of rhythmic properties in connected speech (as reflected in CTenv) is predictive of treatment response in this type of rhythm-based interventions. The current study, therefore, provides a preliminary examination of the relationship between CTenv and treatment response in a rhythm-based treatment described by Quique et al. (2022).

In short, the purpose of this study was threefold. *First*, we examined CTenv in Spanish-speaking PWA and assessed the extent to which CTenv in the delta, theta, and alpha bands was associated with aphasia severity. *Second*, we examined if CTenv in those bands was related to behavioral measures of rhythmic perception and production in PWA. *Third*, we assessed if CTenv in those bands predicted treatment response to the sentence-level rhythm-based intervention previously reported by Quique et al. (2022).

## Methods

### Participants

Nine Spanish-speaking PWA were recruited from an outpatient neurology institute in Medellín, Colombia. These participants were a subset of the Quique et al. (2022) participants, selected because they lived in Medellin, where the necessary EEG instrumentation was located. Inclusionary criteria consisted of aphasia after a single left hemisphere cerebrovascular accident confirmed by neurology referral, a Western Aphasia Battery aphasia quotient between 20 and 85 (WAB-AQ, score range: [0–100]; Kertesz, 1982; Kertesz and Pascual-Leone, 2000), an aphasia post-onset greater than 4 months, and Spanish as a native language. Exclusion criteria consisted of other neurological conditions that could potentially affect speech or cognition, significant premorbid psychiatric history, and severe apraxia of speech. Nine participants were enrolled in the study (Table 1). All participants completed a rhythm-based intervention to learn scripted sentences after their EEG assessment (Quique et al., 2022). Thus, CTenv was assessed at one timepoint, before the rhythm-based intervention. All participants were able to hear pure tones at 40 dB HL at 0.5, 1, 2, and 4 kHz on an audiometric screening test. This study was approved by the institutional review board at the University of Pittsburgh and by the local partner institution in Colombia (Neuromedica).

**Table 1:** Participants’ demographic information.

ID	Sex	MPO	Age	WAB_AQ_	CA-BAT	BAT-sync
COL1	F	12	47	49.1	−2.5	0.93
COL3	F	15	55	55	−0.6	0.92
COL4	F	37	65	65.6	−3.8	0.98
COL6	M	17	53	27.1	−1.4	0.89
COL7	F	24	56	43.7	−1.9	0.79
COL9	M	27	58	20.4	−2.0	0.86
COL10	F	24	42	64.2	−4	0.95
COL12	M	42	31	37.7	−3.5	0.64
COL13	M	14	78	39.5	−2.6	0.84
**Mean**		**23.6**	**53.9**	**44.7**	**−2.5**	**0.9**
**SD**		**10.5**	**13.4**	**15.5**	**1.1**	**0.1**

ID, participant identifier; M, Male, F, Female; MPO, months post-onset of aphasia; Age reported in years; WAB_AQ_, WAB Aphasia Quotient (ranges between 0 and 100); CA-BAT, Perceptual judgment of the beat ability estimate (computed from the underlying item response model, ranges between −4 and 4, approximately); BAT-sync, Correlation between inter-tap mean and target inter-tap intervals adjusting for tactus; SD, standard deviation.

### Sentence-level rhythm-based intervention

Quique et al. (2022) investigated the extent to which unison production of speech could support PWA in learning scripted sentences (i.e., formulaic sentences used in everyday communication). As unison production of speech depends on rhythmic properties, it was hypothesized that highlighting these properties could facilitate learning of scripted sentences. Specifically, Quique and colleagues used rhythmic cues during unison production of speech in which: (a) digitally-added beats were aligned with word stress (stress-aligned condition); (b) digitally-added beats were equally spaced across the sentence (metronomic condition); (c) sentences were presented without added beats (control condition). PWA learned a set of 30 scripted sentences over five training sessions distributed over 2 weeks. As shown in Table 1, participants were between 1 and 3.5 years post-onset of aphasia. PWA demonstrated learning of scripted sentences over time across conditions. Furthermore, both rhythm-enhanced conditions engendered larger treatment response (i.e., improved sentence-level learning) than the control condition.

### Stimuli

**Figure 1:**
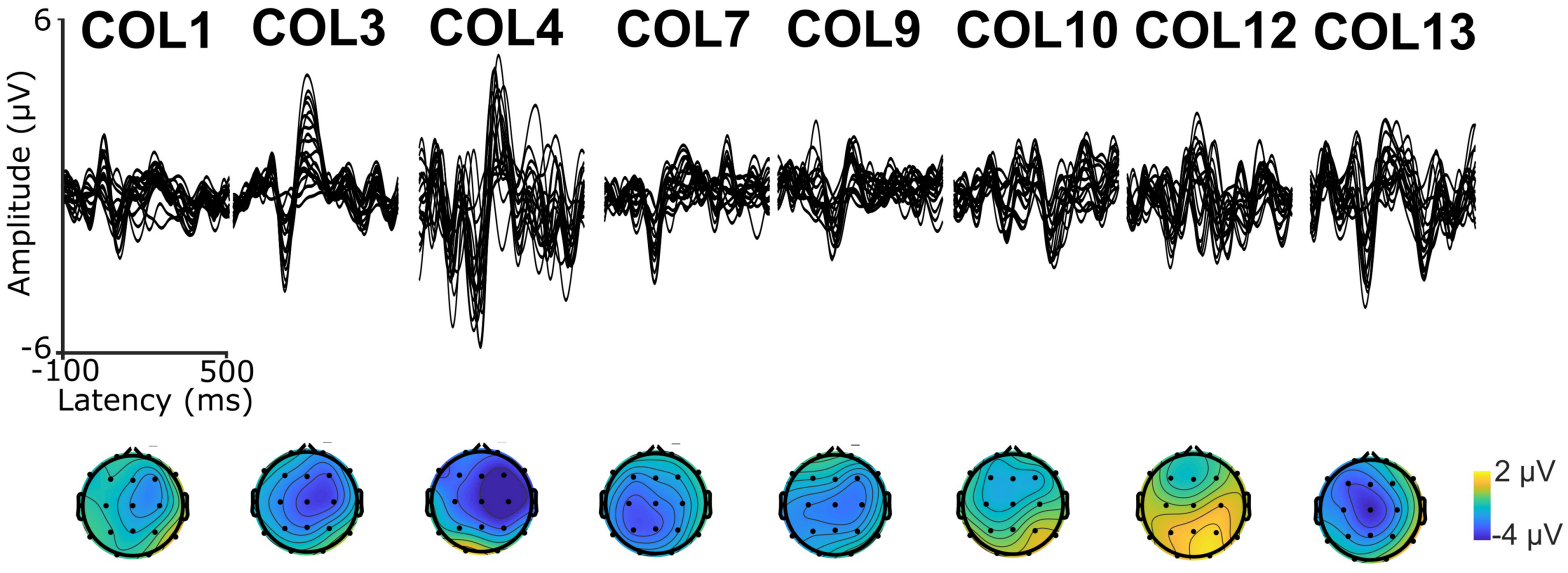
Auditory Long Latency Responses (ALLRs, top) and scalp topography (bottom) of the first negative wave (N1) in each participant.

A 9-min excerpt of a Spanish adaptation of the audiobook “Who was Albert Einstein” (Brallier, 2002) was used to assess CTenv. The excerpt was recorded by a human male native speaker of Spanish. The excerpt was divided into approximately 1-min segments, each beginning and ending with complete sentences to preserve the storyline. To ensure attention, participants answered a comprehension question after each segment, as previously used in PPA (Dial et al., 2021). See Supplementary material for an example of a story segment. The envelope spectrum of the excerpt was 6.3 Hz, which is closely related to the syllable rate (Obleser and Weisz, 2012; Ghitza, 2013; Kösem and van Wassenhove, 2016). See modulation spectrum in Supplementary Figure S1. Also, to validate the recording of auditory electrophysiology responses and ensure that auditory stimuli reached the auditory cortex, we recorded Auditory Long Latency Responses (ALLRs; Swink and Stuart, 2012; Reetzke et al., 2021; See Figure 1). For this purpose, we used a sinusoidally modulated (6 Hz) tone of carrier frequency of 1,000 Hz and another unmodulated pure tone of frequency of 1,000 Hz. Both tones were 1 second in duration and were repeated 100 times, with an inter-stimulus interval of 1 second.

### Instrumentation

A USB audio interface (Behringer U-Phoria*–*UMC404HD) was used to present the auditory stimuli. Electroencephalography (EEG) was performed using a Nihon*–*Kohden 32*–*channel clinical EEG system. Nineteen scalp electrodes were placed on the scalp following the international 10*–*20 system (Klem et al., 1999), along with two mastoid electrodes. The online reference was placed at Cz. Electrode impedance was under 5 kΩ for most electrodes, and the maximum impedance did not exceed 20 kΩ. The sampling rate for EEG recording was 5,000 Hz. The data were re-referenced offline to the average of both mastoids. The analog electrical audio signal from one of the audio interface outputs was directly plugged into one of the leads on the EEG amplifier (with appropriate attenuation) to align the EEG to the stimulus onset. Further, as the EEG system was used for clinical purposes, ALLRs were recorded to ensure that the instrumentation was optimal. Specifically, if the instrument was sensitive to record reliable ALLRs in the latency range of 50 to 250 ms that were higher than the pre-stimulus baseline and had a morphology and topography consistent with ALLRs.

### Procedure

Participants were first presented with the ALLRs stimuli and then listened to the audiobook. Stimuli were presented binaurally with insert earphones (ER-3A; Etymotic Research) at 84 dB SPL (Becker and Reinvang, 2007; Robson et al., 2014) using PsychoPy (Peirce et al., 2019). During ALLRs, participants were instructed to listen to the tones without any additional task. During the audiobook, participants were instructed to listen to the story and answer the multiple-choice questions. They were also asked to avoid extraneous movements and look at a fixation cross on the computer screen.

### EEG processing

Preprocessing was performed using EEGLAB (Delorme and Makeig, 2004) in MATLAB (MathWorks Inc., 2019). To reduce data size and improve computational efficiency, we first down-sampled the data to 128 Hz. Then, we applied minimum-phase non-causal Sinc FIR filters with a high pass cut-off frequency of 1 Hz, filter order of 846; and a low-pass cut-off frequency of 15 Hz, filter order of 212. Filtered data were then re-referenced to the two mastoid channels (Di Liberto et al., 2015; O’Sullivan et al., 2017). Large movement artifacts were suppressed using Artifact Subspace Reconstruction (ASR; Mullen et al., 2013; Plechawska-Wojcik et al., 2019). After ASR, the data were separated into epochs from −5 s to 70 s (i.e., the onset of each 1-min segment), resulting in 9 epochs. Independent Component Analysis was performed on the epoched data to remove eye movement and muscle artifacts (Jung et al., 2001). The independent components were then inspected visually for time course, topography, and spectrum and were removed if they corresponded to ocular or muscular activity.

To extract the theta, and alpha bands from the EEG, the data was further filtered using third-order butterworth filters; theta (lf = 4 Hz; hf = 8 Hz), and alpha (lf = 8 Hz; hf = 15 Hz). For the delta however, a different preprocessing procedure was followed, where the high pass filter was changed. The raw EEG data was filtered using a windowed sinc filters (lf = 0.75, order 846; hf = 15, order 212). This data was subjected to the same preprocessing steps until ICA. The ICA weights that were derived from the 1–15 hz filtered data were copied over to 0.75–15 Hz filtered data (Barbieri et al., 2021). The ICA components with artifacts were then rejected. This was done to ensure good ICA decomposition, as highpass filtering with 1 Hz provides better stationarity in the EEG (Winkler et al., 2015). Finally, to extract the delta band, this data was low pass filtered at 4 Hz with a third-order butterworth filter which essentially resulted in a 0.75–4 Hz filter band. The magnitude response of the filters and filter characteristics used to derive the three different EEG bands are shown in Supplementary Figure S2.

### EEG analysis

**Figure 2:**
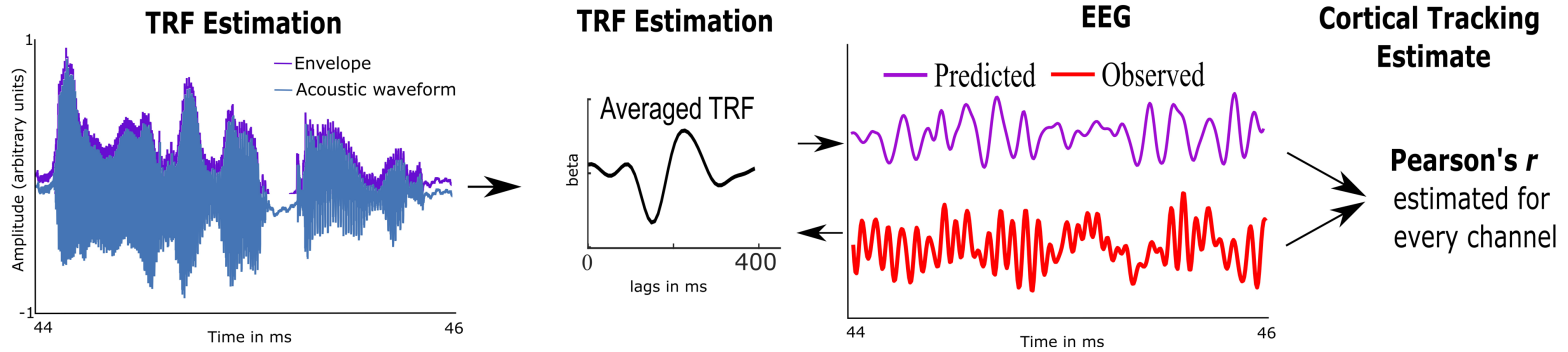
Schematic representation of the Temporal Response Function (TRF), used to estimate cortical tracking of the speech envelope (CTenv; mapping of speech envelope to the neural data). A linear kernel to map the speech envelope of the audiobook onto the EEG is estimated using ridge regression. The cross-validated model fit is estimated using Pearson’s correlation between the EEG-predicted data and the observed EEG, which serves as a metric of CT.

Preprocessed EEG was analyzed using custom routines in Matlab and the multivariate Temporal Response Function toolbox (mTRF; Crosse et al., 2016). We used mTRFs with forward modeling to estimate how well the temporal envelope predicted EEG activity; this provided an individual measure of CTenv (McHaney et al., 2021). First, the multiband Hilbert transform was used to estimate the temporal envelopes of each 1-min speech segment. Second, using the preprocessed EEG data and the temporal envelopes, the mTRFs were estimated *via* regularized linear ridge regression (see Figure 2 and Supplementary Figure S3). mTRFs were estimated for lags ranging from −100 ms to 650 ms. To reduce overfitting and obtain the best model fit across electrodes and participants, a regularization parameter optimized from 2^0^ to 2^20^ using *k*-fold (leave-one-out) cross-validation was applied (Crosse et al., 2016). Model fit was assessed using Pearson’s correlation between the predicted EEG (stimulus envelope convolved with mTRFs) and the observed EEG data, which was considered the CTenv estimate. To test the robustness of the obtained CTenv estimates, we compared them against random CTenv estimates generated from one hundred mismatched permutations of EEG responses and the audiobook envelopes. Comparing the observed CTenv against the random CTenv provides evidence regarding the association between cortical activity due to specific auditory input in the speech envelope rather than a chance response. As CTenv can be affected by the cortical lesions and related volume conducted electrical field at the electrodes, only the five electrodes that had the highest CTenv per participant were averaged to obtain one metric of cortical tracking.

### Behavioral measures

To characterize *language performance*, participants were given the Spanish adaptation of the Western Aphasia Battery (WAB; Kertesz, 1982; Kertesz and Pascual-Leone, 2000). To characterize *rhythmic perception and production*, participants were given the computerized version of the Beat Alignment Test (CA-BAT; Harrison and Müllensiefen, 2018) and the Synchronization with the Beat of Music Task from the Beat Alignment Test (BAT-sync; Iversen and Patel, 2008). In the CA-BAT, participants listen to musical excerpts presented twice: once with superimposed beats arranged in synchrony with the excerpt and once with beats either too fast or too slow in relation to the excerpt. Participants are asked to indicate in which of the two presentations the beats were “on the beat” with the excerpt. In the BAT-sync, participants are asked to listen and tap with their left hand to the beat of 12 musical excerpts with various musical styles. The BAT-sync provides a measure of synchronization (ranging between 0 and 1) based on the correlation between intertap means (average tapping intervals for each of the 12 excerpts) and tactus levels of the underlying beat of each excerpt (most likely to occur tapping tempos).

### Variables and statistical analysis

Statistical analyzes were performed in Rstudio (RStudio Team, 2016; R Core Team, 2020). We used a robust bayesian estimation to assess if the observed CTenv differed from an empirical chance distribution (see Kruschke, 2013). To examine the three study aims, we implemented bayesian generalized linear regression models using Stan (Carpenter et al., 2017; Stan Development Team, 2020) with the R brms package (Bürkner, 2018). This modeling approach is advantageous for the exploratory nature of the current study as it characterizes uncertainty in the estimates of interest through credible intervals (Kruschke and Liddell, 2018), rather than *p*-values, and quantifies whether the data support hypothesized effects or null findings.

The association between *CTenv and aphasia severity* was assessed using WAB-AQ as the dependent variable and CTenv estimates in the alpha, delta, and theta bands as predictors. The association between *CTenv and rhythmic perception/production measures* was assessed using two models, one for each rhythmic measure (CA-BAT and BAT-sync), with CTenv estimates in the alpha, delta, and theta bands as predictors. The association between *CTenv and treatment response to the rhythm-based intervention* from Quique et al. (2022) was assessed using mixed-effects models to take advantage of the available trial-level treatment data. Specifically, trial-level word accuracy (i.e., the proportion of correct words out of the total number of words in each sentence, at each session). was used as a dependent variable. Fixed effects were session and CTenv estimates. Random effects included random intercepts for participants and items, and a random slope of session for participants. Models were run separately for the rhythm-enhanced and control conditions. Details about each model can be found in Supplementary materials.

## Results

**Figure 3:**
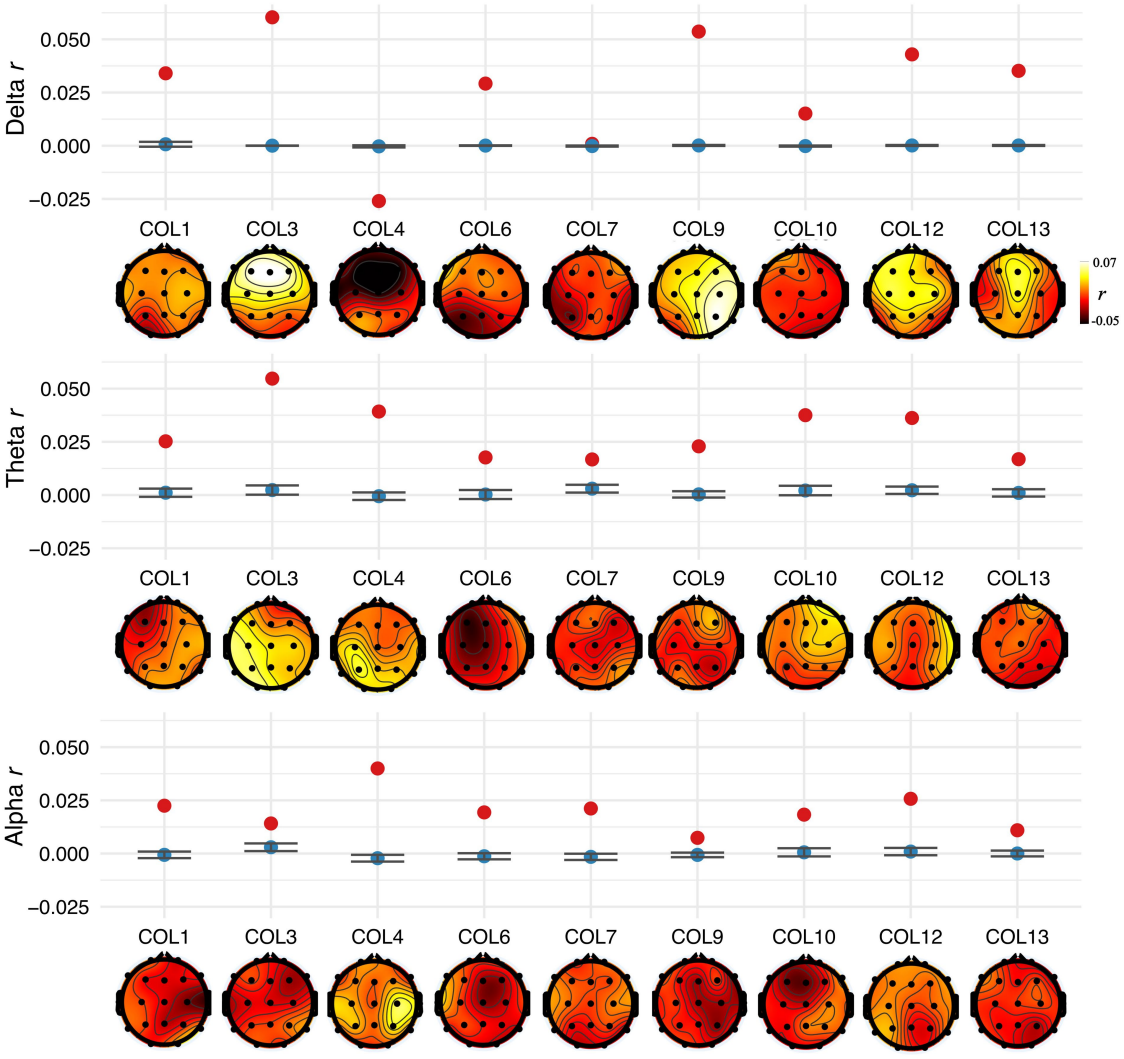
Observed (averaged across the five best electrodes) CTenv (blue) and CTenv to null/random model (red; based on 100 random pairings of the speech envelope and EEG) in each participant for the delta, theta, and alpha band, respectively. Error bars represent 95% confidence intervals of chance distribution. The scalp topography of CTenv estimates (Pearson’s correlation) in the delta, theta, and alpha bands, are shown at the bottom of each panel for each participant.

Nine PWA were enrolled and completed the study. All participants showed ALLR peaks, indicating that: (1) the EEG setup was appropriate to record neural responses evoked by auditory signals, and (2) the auditory neural centers in all the participants received afferent input regarding sound onset information. See Figure 1 for a depiction of ALLRs per participant. Thus, we assessed CTenv in all the participants. The strength of CTenv estimates was consistent with previous reports (Di Liberto et al., 2015). Based on 90% credible intervals, the observed CTenv estimates were reliably higher than the randomly generated CTenv estimates across bands and participants (ß = −0.03, CI: −0.03, −0.02), indicating an association between cortical processing and a specific auditory input (i.e., the speech envelope). See Figure 3 for a depiction of observed versus random CTenv measures per participant and band.

### Association between CTenv and aphasia severity

**Figure 4:**
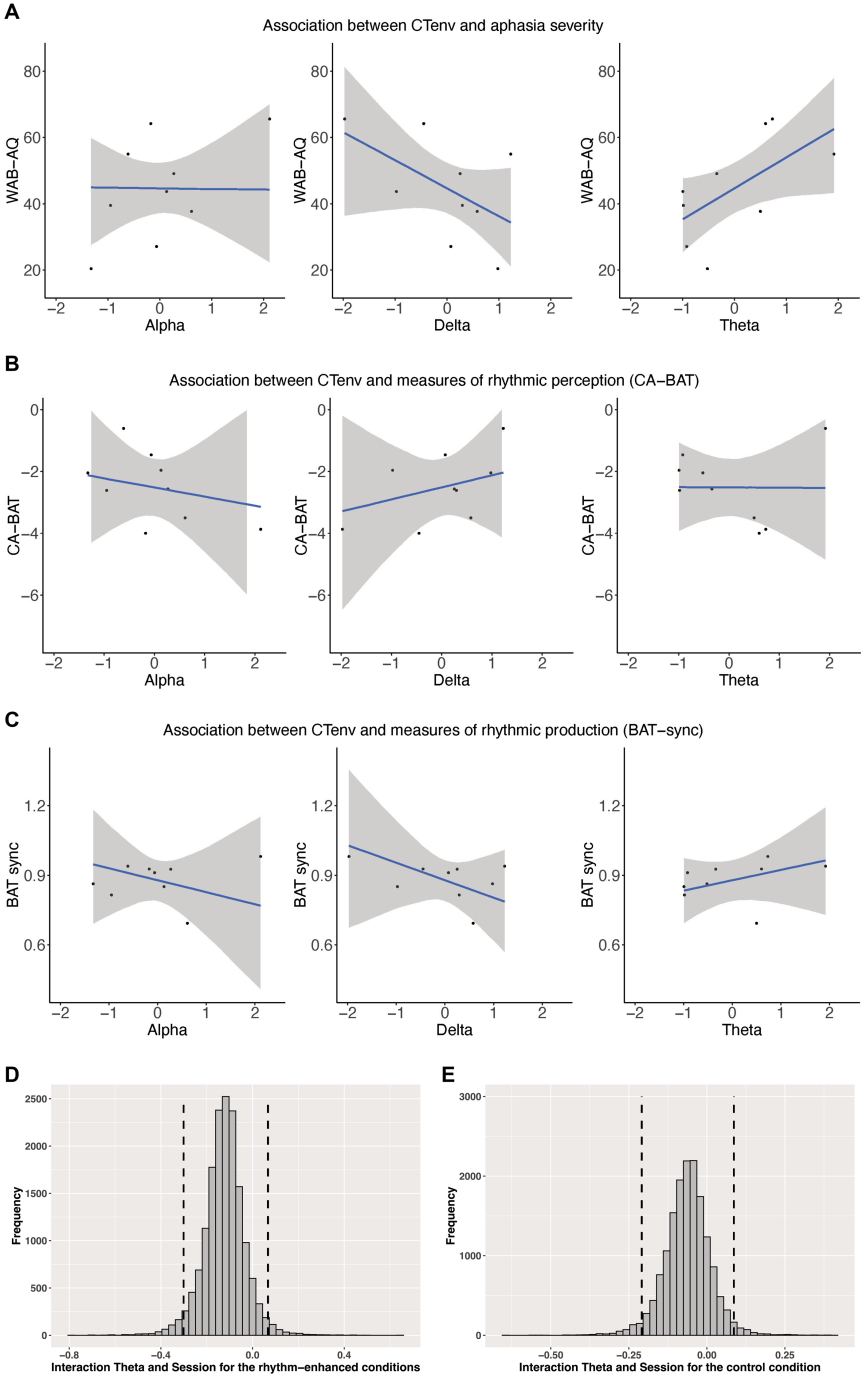
**(A)** Shows scatterplots of the association between CTenv and aphasia severity. **(B)** Shows Scatterplots of the association between CTenv and CA-BAT. **(C)** Shows Scatterplots of the association between CTenv and BAT-sync. Black dots = raw data. Blue line = estimated slope from models reported in Supplementary Tables S1–S3. Gray area = 90% model uncertainty intervals. **(D,E)** Show the posterior distribution and 90% highest density intervals (HDIs) of the interaction effects between the theta band and session for the rhythm enhanced and control conditions. Dashed lines mark the 90% highest density intervals (HDIs) for the posterior distribution.

We assessed whether CTenv measures could be used as a metric to predict aphasia severity. CTenv in the theta band was a reliable predictor of aphasia severity (ß = 9.07, CI: 1.94, 15.29), suggesting that increased tracking in this band is associated with an increase in WAB-AQ (less impairment). The credible interval for CTenv in the delta band included zero (ß = −8.06, CI: −16.27, 1.48), and so did not meet the criteria we defined as a robust effect, but 93% of the posterior distribution for this effect was below zero. This provides weak but positive evidence that increased tracking in the delta band could be associated with a decrease in WAB-AQ (more impairment). CTenv in the alpha band was not a reliable predictor of aphasia severity (ß = 0.07, SE = CI: −8.26, 9.25). See Figure 4. See also Supplementary Figure S4 in our Supplementary materials.

### Association between cortical tracking and measures of rhythmic perception and production

CTenv did not reliably predict CA-BAT performance (delta: ß = 0.39, CI: −0.84, 1.63; theta: ß = −0.01, CI: −0.91, 0.87; alpha: ß = −0.29, CI: −1.53, 0.99). Similarly, CTenv did not reliably predict BAT-sync performance (delta: ß = −0.07, CI: −0.20, 0.06; theta: ß = 0.04, CI: −0.04, 0.13; alpha: ß = −0.05, CI: −0.18, 0.08). These results indicate little association between rhythm perception/production (as measured by CA-BAT and BAT-sync) with CTenv in PWA. See Figure 4. See also Supplementary Figures S5, S6 in our Supplementary materials.

### Association between CTenv and a rhythm-based intervention

CTenv in the theta band showed a reliable main effect for the rhythm-enhanced conditions (ß = 1.06, CI: 0.48, 1.66), and a similar effect for the control condition (ß = 0.80, CI: 0.01, 1.59). Thus, these results indicate that the odds of producing a correct word, at the midpoint of treatment, were greater in people with higher CTenv in the theta-band. Consistent with Quique et al. (2022), within this sub-group of participants from Quique et al. (2022), there was also a reliable main effect of session for all models across conditions and bands (all estimates were positive, and the CI excluded zero), indicating a positive treatment response over time. Although the interaction effect between CTenv in theta and session for the rhythm-enhanced (ß = −0.12, CI: −0.27, 0.02) and control conditions (ß = −0.06, CI: −0.18, 0.05) included zero, examining the posterior distributions showed indications of a positive effect. Specifically, 93% of the posterior distribution was below zero for the rhythm-enhanced, and 83% for the control conditions (See Figure 4). This result provides weak but positive evidence that a higher CTenv in theta may be associated with a reduced treatment response to the sentence-level rhythm-based intervention. There were no other main effects or interaction effects for CTenv in alpha or delta bands (CI included zero with no substantial portions of their posterior distribution suggesting meaningful effects). See Supplementary materials for complete model output for all models (Supplementary Figures S7–S16).

## Discussion

The current study examined CTenv in Spanish speakers with aphasia and its association with language impairments, behavioral measures of rhythmic perception/production, and treatment response to a rhythm-based intervention. CTenv in the theta band was associated with language impairments in our cohort of Spanish speakers with stroke-induced aphasia. We also found some evidence that CTenv in the delta band may be associated with language impairments. These preliminary findings may suggest that cortical activity in the theta and delta bands, which have been shown to be essential for speech processing in neurotypical individuals (Poeppel and Assaneo, 2020), could be affected by acquired brain lesions.

The direction of the relationship between language impairments and CTenv in the delta and theta bands had intriguing patterns. Higher CTenv in the delta band was likely associated with more-severe language impairments, whereas higher CTenv in theta band was associated with less-severe language impairments. Although this opposite directionality could indicate an inverse relationship between delta and theta rhythms to compensate for language impairments in aphasia, our results do not provide conclusive evidence. The association between higher CTenv in the delta band and more severe language impairments could reflect a compensatory mechanism to support language comprehension. Such increases in CTenv and decreased speech comprehension have been reported in older adults, suggesting that increased tracking could indicate altered speech processing abilities (e.g., Decruy et al., 2019). McHaney et al. (2021) reported an association between increased delta tracking and speech-in-noise comprehension in older adults (for speech in noise compared to quiet). However, other studies have shown that increased CTenv may reflect, instead, an imbalance between excitatory and inhibitory brain processes or the recruitment of additional brain regions for processing information (e.g., Brodbeck et al., 2018; Zan et al., 2020).

The association between CTenv in the theta band and language impairments could suggest that the higher CTenv, the more appropriately PWA can segment syllabic information (Teng et al., 2018). However, increased tracking in the theta band has been observed in individuals with logopenic PPA, who typically show phonological processing deficits, compared to neurotypical older adults (Dial et al., 2021). Dial et al. (2021) suggested that increased tracking in PPA could result from a shift of function from atrophic temporoparietal cortex to spared brain regions in both hemispheres; it could be further affected by increased functional connectivity in the temporal cortices and hypersynchrony in the frontal cortex (Ranasinghe et al., 2017). Such a shift of function could also be seen in brain damage due to stroke; however, the pathology differs from PPA. Further, Etard and Reichenbach (2019) reported that increased tracking in the theta band was associated with perceived speech clarity in young adults. Given the lack of evidence in CTenv and aphasia, or other acquired neurological disorders, these interpretations need to be investigated in future studies. The exact mechanism driving the relationship between cortical tracking and aphasia severity needs further large-scale intervention across a wide range of aphasia severities using spatially and temporally precise neuroimaging approaches.

None of the CTenv bands were reliable predictors of behavioral measures of rhythm perception (i.e., CA-BAT, BAT-sync); this lack of association is inconsistent with previous findings. For example, Nozaradan et al. (2011) found that in neurotypical participants, beat perception was associated with a sustained periodic response in the EEG spectrum. Similarly, Nozaradan et al. (2013) showed links between neural responses, listening to rhythmic sound patterns, and tapping to beats. Further, Colling et al. (2017) showed that CTenv and beat synchronization to a metronome were affected in individuals with dyslexia. Although we did not simultaneously use EEG with behavioral measures of rhythm perception/production, given previous evidence, an association would have been expected. Thus, more research is needed to understand associations between CTenv and measures of rhythm perception/production in acquired neurological disorders.

The preliminary interaction effect between CTenv in theta and session indicates that there may be an association between CTenv and treatment response to a sentence-level rhythm-based intervention, but this conclusion would require additional examination and replication. Rhythm-based interventions have been recommended in language disorders such as dyslexia (e.g., Goswami, 2011; Thomson et al., 2013), which has been linked to concomitant rhythmic difficulties (e.g., Wolff, 2002; Flaugnacco et al., 2014; Ladányi et al., 2020). Similarly, individuals with aphasia have shown rhythm processing deficits (Zipse et al., 2014; Sihvonen et al., 2016), but have also benefited from rhythm-based interventions (e.g., Stahl et al., 2011, 2013; Quique et al., 2022). Further research can continue to examine whether encoding of speech rhythmic properties could provide neurophysiologically informed decision-making regarding the choice of intervention in individuals with aphasia.

## Limitations and future work

The current study had some limitations. First, it had a limited sample size of PWA; thus, reproducing our findings in future studies with larger sample sizes would be needed to provide robust evidence. Second, there were limited observations within each variable available for modeling (e.g., for aphasia severity and rhythm perception/production, we had one observation per participant). Thus, replicating these findings in larger samples with multiple observations per participant is necessary. Third, because of the cortical lesions in our participants with aphasia, we did not select electrodes of interest *a priori*; instead, we used the averaged CTenv of the five electrodes with the highest values. However, future studies should acquire anatomical data and perform source-level analysis to avoid confounds of volume conduction artifacts introduced by the brain lesion. Similarly, we believe that anatomical changes in the cortex after stroke may have an influence on CTenv, but we did not have lesion data for these participants; this information is needed to investigate the role of lesion size and location on CTenv in aphasia. Fourth, our results could not provide a clear mechanistic explanation for the directionality of the relationship between CTenv and aphasia severity. Future studies are necessary to understand the neurophysiological mechanisms underlying such findings. Fifth, CTenv was assessed only at one timepoint (before the rhythm-based intervention). Future studies using such neural metrics before and after intervention could provide objective neurophysiological evidence that can guide interventions, predict outcomes, and measure the mechanisms of treatment-related improvements. Overall, CTenv in the theta band was associated with language impairments. CTenv in theta may be associated with treatment response to sentence-level rhythm-based interventions, but further research is needed to provide robust evidence.

## Data availability statement

The raw data supporting the conclusions of this article will be made available by the authors, without undue reservation.

## Ethics statement

The studies involving humans were reviewed and approved by the Institutional Review Board at the University of Pittsburgh and by Neuromedica, the local partner institution in Colombia. The studies were conducted in accordance with the local legislation and institutional requirements. The participants provided their written informed consent to participate in this study.

## Author contributions

Data collection, analysis, and background literature review were conducted by YQ, GNG, WE, and MD. Data analyses and interpretation were conducted by YQ and GNG. Manuscript preparation was conducted by YQ, GNG, WE, MD, and BC. All authors contributed to the article and approved the submitted version.

## Funding

This research project was funded by the Council of Academic Programs in Communication Sciences and Disorders (CAPCSD) Ph.D. Scholarship, the ASHFoundation New Century Scholars Doctoral Scholarship, and the Doctoral Student Award from the University of Pittsburgh School of Health and Rehabilitation Sciences to YQ. Funding to BC was by National Institute for Communication Sciences and Disorders award R01DC013315 and Northwestern University. Funding to GNG was provided by the Vice-Chancellors Graduate Research Education funds.

## Conflict of interest

The authors declare that the research was conducted without any commercial or financial relationships that could be construed as a potential conflict of interest.

## Publisher’s note

All claims expressed in this article are solely those of the authors and do not necessarily represent those of their affiliated organizations, or those of the publisher, the editors and the reviewers. Any product that may be evaluated in this article, or claim that may be made by its manufacturer, is not guaranteed or endorsed by the publisher.
