# Supplementary material for: Examining cortical tracking of the speech envelope in post-stroke aphasia
Source: Front Hum Neurosci. 2023 Sep 14;17:1122480. doi: 10.3389/fnhum.2023.1122480 (PMC10538638; doi:10.3389/fnhum.2023.1122480)
Supplement: Supplementary file 1 [file Data_Sheet_1.docx]

**SUPPLEMENTAL MATERIAL**

Example of the first story segment of the Spanish adaptation of the book “Who was Albert Einstein” (Brallier, 2002), and its associated question. Please note that for copyrights, we only show one segment of the stort.

**First segment (Spanish version)**

Alber Einstain llegó al mundo el 14 de marzo de 1879 en Ulm, Alemania. Por cierto que no parecía ser un niño extraordinario, era regordete y pálido, y tenía el pelo negro y abundante. Era tan callado y tímido que sus padres llegaron a pensar que tenía algún problema. Lo llevaron a varios médicos. “No habla,” decían sus padres. Los médicos nunca encontraron nada malo en él. Según cuentan, Albert no dijo ni una sola palabra hasta los tres o cuatro años de edad. Derrepente una noche, mientras cenaban, habló. “La sopa está demasiado caliente,” dijo. Sus padres aliviados le preguntaron porqué no había dicho nada antes. “Porque hasta ahora todo ha estado bien,” contestó el pequeño Albert. No hay pruebas sin embargo de que esta historia sea cierta. La mayoría de los niños de su edad jugaban a los soldados y otros juegos bruscos y violentos, pero Albert no.

**First segment (English version)**

Albert Einstein made his entrance into the world on March 14, 1879, in Ulm, Germany. He certainly didn´t seem like an extraordinary child. He was chubby and pale with thick, black hair he was so quiet and shy that his parents worried that there was something wrong with him. They took Albert to doctors. “He doesn´t talk,” his parents explained. The doctors found nothing wrong. The story goes that albert didn’t speak a word until he was three or four years old. The suddenly, over supper one night, he said, “The soup is too hot.” Greatly relieved, his parents asked why he had never said anything before. “Because,” little Albert replied, “up to now, everything has been fine,” Is this story true? There’s no proof. Most boys his age played solder and other rough-and-tumble games. Not Albert.

*Question for the first segment* ***(Spanish version)***

¿En qué cuidad nació Albert Einstein?

1. Ulm
2. Berlín
3. Frankfurt
4. Weimar

*Question for the first segment* ***(English version)***

In which city was Albert Einstein born?

1. Ulm
2. Berlin
3. Frankfurt
4. Weimar


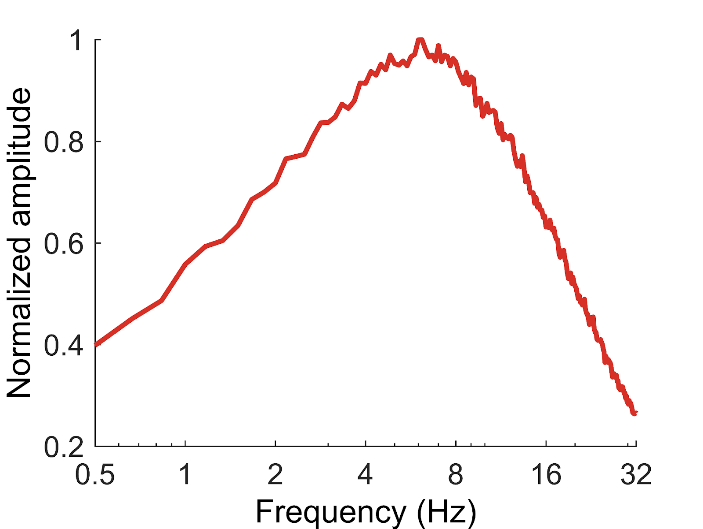


**Figure S1.** Mean modulation spectrum of the audiobook (based on Ding et al., 2017) used in the current study shows a peak modulation of ~6.3 Hz.

**
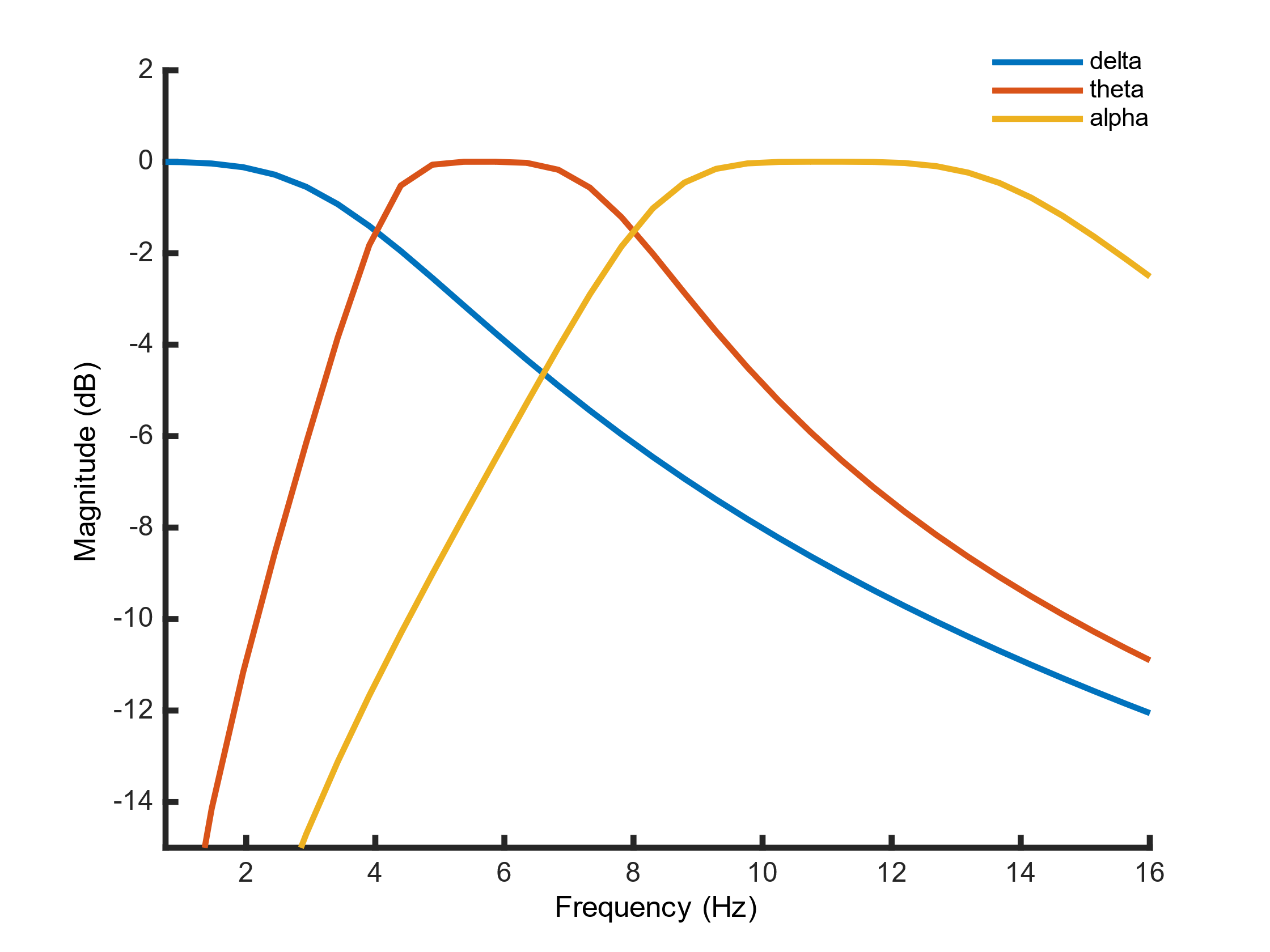
**

**Figure S2.** Magnitude responses of the EEG filters used to separate the EEG bands.

**
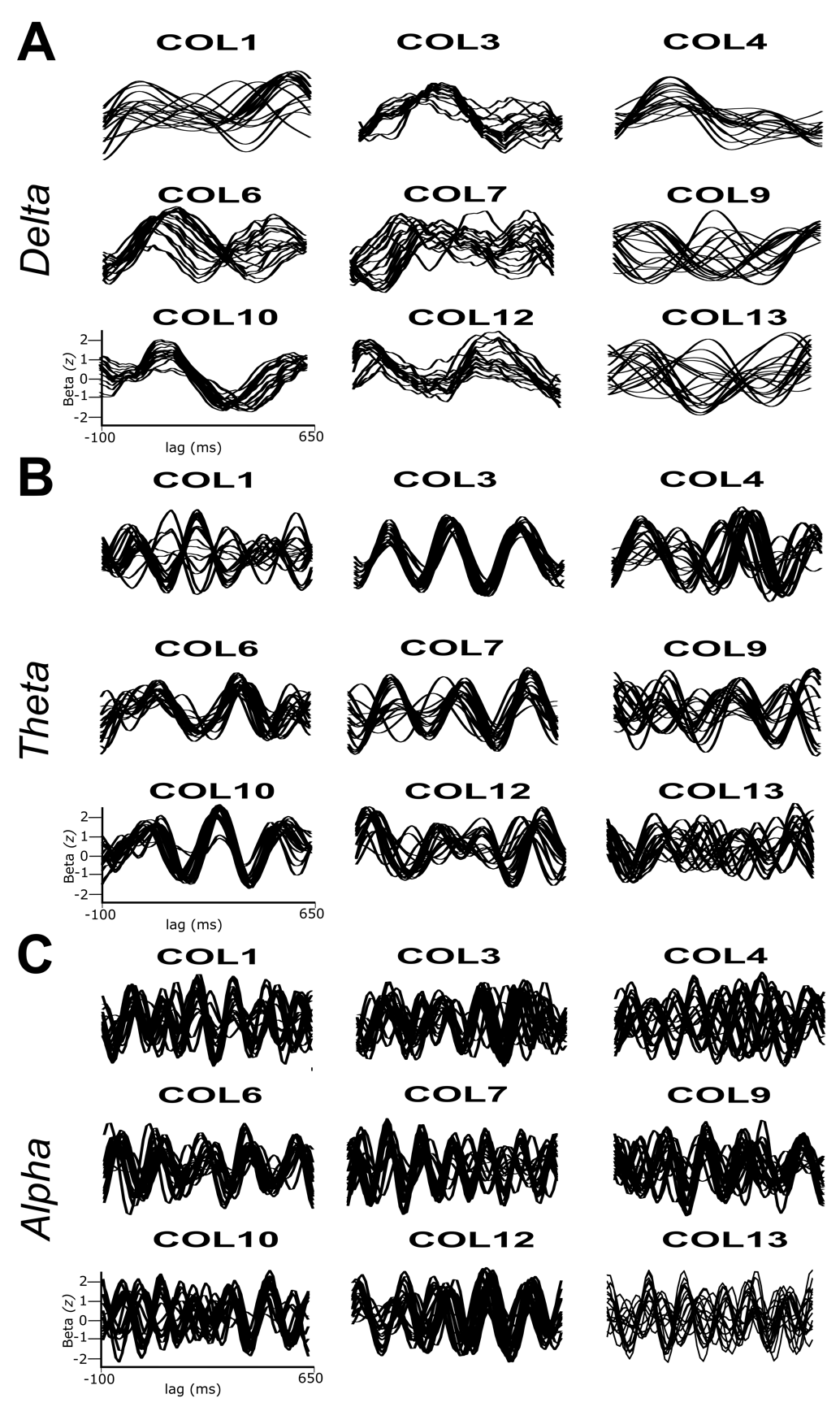
**

**Figure S3.** A, B, and C show TRFs, where each trace is a single electrode in the delta, theta, and alpha bands, respectively.

We used Bayesian generalized regression models to analyze each of the three aims. For the model of association between CTenv and aphasia severity, we included weakly informative priors for all beta coefficients using a normal distribution (mu = 0, and sigma = 13). For the model of association between cortical tracking and measures of rhythmic perception (CA-BAT), we included weakly informative priors for all beta coefficients using a normal distribution (mu = 0, and sigma = 2). For the model of association between cortical tracking and measures of rhythmic production (BAT-sync), we included weakly informative priors for all beta coefficients using a normal distribution (mu = 0, and sigma = 1). We used default BRMS priors for the variance term in all models.

Predictor variables were z-scored before inclusion in the models. For all models, we used four Hamiltonian Markov chain Monte Carlo (MCMC) chains, each starting at random initial values. Each chain consisted of 3,000 iterations with 2,000 initial warm-up iterations, which were not used for parameter estimation. We assessed model convergence and healthy mixing of the MCMC chains using the Gelman-Rubin Potential Scale Reduction statistic ($\hat{R}$), the number of effective samples, and trace plots. Specifically, we verified that $\hat{R}$ values were below 1.01, that each parameter estimate had a number of effective samples greater than 400, and that trace plots showed good mixing. We have provided $\hat{R}$ and the number of effective samples for each model in the tables below. Also, we assessed model fit with posterior predictive checks, observing that the distributions of predicted parameters approximated the observed data distribution. We have also provided posterior predictive figures below.

**Table S1. Association between CTenv and aphasia severity**

|  | **Estimate** | **SE** | **90% CI** | $\hat{\boldsymbol{R}}$ | **Bulk ESS** | **Tail ESS** |
| --- | --- | --- | --- | --- | --- | --- |
| Intercept | 44.66 | 3.74 | 38.63, 50.8 | 1 | 10660 | 8288 |
| Alpha | 0.07 | 5.36 | -8.26, 9.25 | 1 | 6650 | 8244 |
| Delta | -8.06 | 5.39 | -16.27, 1.48 | 1 | 6576 | 7603 |
| Theta | 9.07 | 4.11 | 1.94, 15.29 | 1 | 7929 | 7623 |
| brms model statement: WAB_AQ_ ~ alpha + delta + theta | | | | | | |

*Notes:* SE = Standard Error. ESS = Effective Sample Size. $\hat{R}$ = Gelman-Rubin potential scale reduction statistic. CI = Credible Interval. CTenv in the alpha, delta, and theta were z-scored.

**
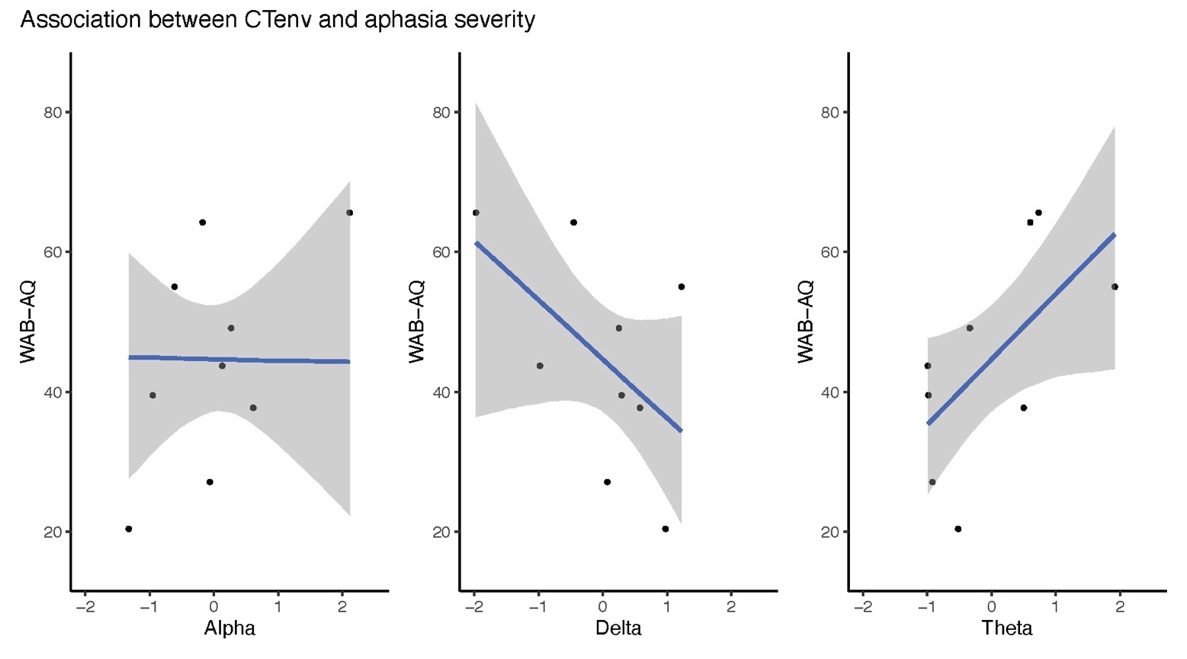
**

**Figure S4.** Scatterplots of the association between CTenv and aphasia severity. Black dots = raw data. Blue line = estimated slope from model reported in table S1. Gray area = 90% uncertainty intervals from the model reported in table S1.

**Table S2. Association between cortical tracking and measures of rhythmic perception (CA-BAT)**

|  | **Estimate** | **SE** | **90% CI** | $\hat{\boldsymbol{R}}$ | **Bulk ESS** | **Tail ESS** |
| --- | --- | --- | --- | --- | --- | --- |
| Intercept | -2.52 | 0.46 | -3.26, -1.79 | 1 | 10525 | 8297 |
| Alpha | -0.29 | 0.77 | -1.53, 0.99 | 1 | 6695 | 8155 |
| Delta | 0.39 | 0.76 | -0.84, 1.63 | 1 | 7253 | 8131 |
| Theta | -0.01 | 0.55 | -0.91, 0.87 | 1 | 8602 | 8777 |
| brms model statement: CA-BAT ~ alpha + delta + theta | | | | | | |

*Notes:* SE = Standard Error. ESS = Effective Sample Size. $\hat{R}$ = Gelman-Rubin potential scale reduction statistic. CI = Credible Interval. CTenv in the alpha, delta, and theta were z-scored.


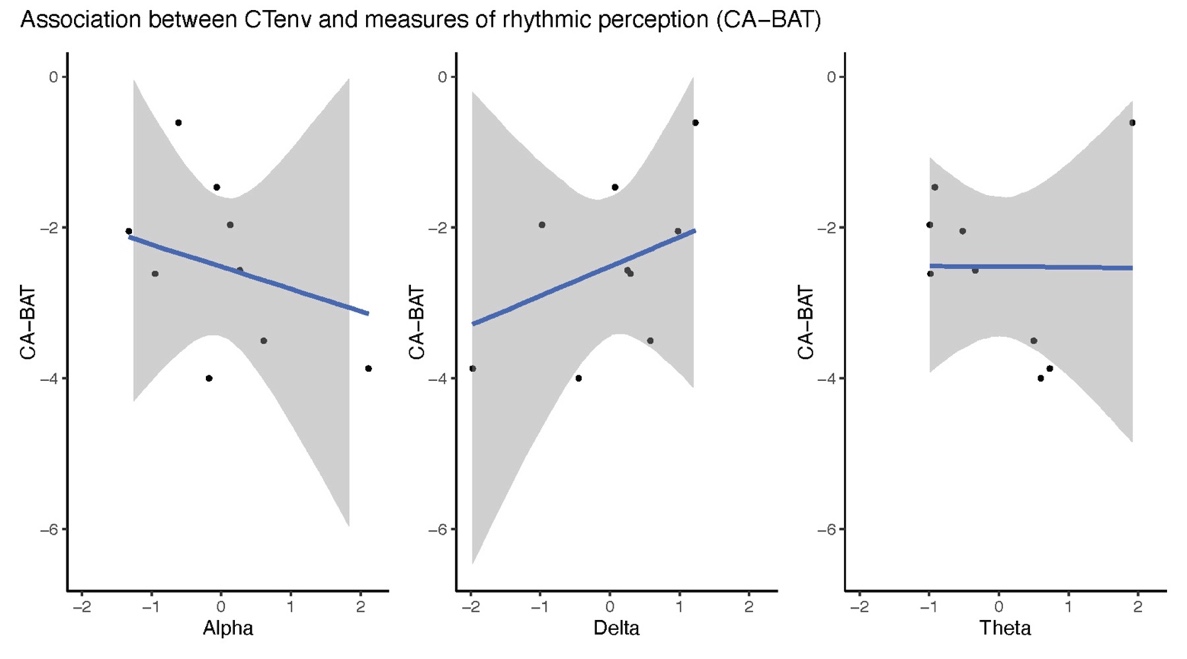


**Figure S5.** Scatterplots of the association between CTenv and CA-BAT. Black dots = raw data. Blue line = estimated slope from model reported in table S2. Gray area = 90% uncertainty intervals from the model reported in table S1.

**Table S3. Association between cortical tracking and measures of rhythmic production (BAT-sync)**

|  | **Estimate** | **SE** | **90% CI** | $\hat{\boldsymbol{R}}$ | **Bulk ESS** | **Tail ESS** |
| --- | --- | --- | --- | --- | --- | --- |
| Intercept | 0.88 | 0.04 | 0.81, 0.95 | 1 | 9258 | 7469 |
| Alpha | -0.05 | 0.09 | -0.18, 0.08 | 1 | 6081 | 5721 |
| Delta | -0.07 | 0.08 | -0.20, 0.06 | 1 | 6053 | 6224 |
| Theta | 0.04 | 0.06 | -0.04, 0.13 | 1 | 6929 | 6985 |
| brms model statement: BAT-sync ~ alpha + delta + theta | | | | | | |

*Notes:* SE = Standard Error. ESS = Effective Sample Size. $\hat{R}$ = Gelman-Rubin potential scale reduction statistic. CI = Credible Interval. CTenv in the alpha, delta, and theta were z-scored.


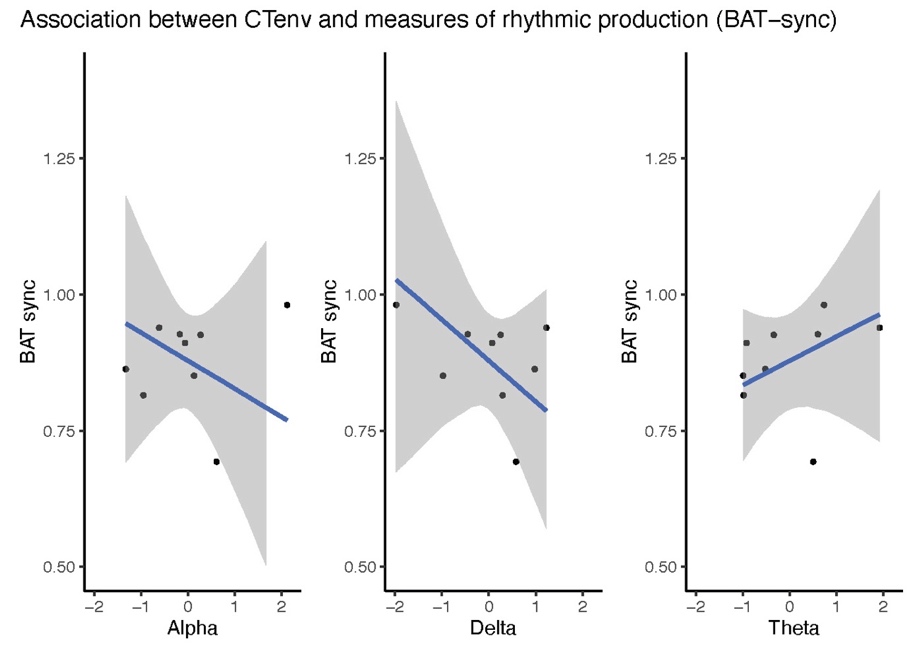


**Figure S6.** Scatterplots of the association between CTenv and BAT-sync. Black dots = raw data. Blue line = estimated slope from model reported in table S3. Gray area = 90% uncertainty intervals from the model reported in table S1.

In each of the following six models, we used Bayesian hierarchical generalized linear models with a logistic–binomial link function. For each model, we included weakly informative priors for all beta coefficients using a normal distribution (mu = 0, and sigma = 5). For the variance term, we used a cauchy distribution (mu = 0, and sigma = 5). Each model, included fixed effects for session and CTenv estimates. The fixed effect of session corresponds to the five training sessions that participants received in the intervention reported in Quique et al. (2022). The by-subject random intercept allows participants to start at different accuracy levels at the beginning of the intervention, and the by-subject random slope for session allows participants to change performance at different rates over time (1+session|participant). The by-items random intercept allows individual sentences being learned by participants to vary in their starting accuracy (1|sentence). Also, we added an observation-level (1|obs) random effect to account for overdispersion, as suggested by Gelman and Hill (2006).

**Table S4. Alpha (rhythm-enhanced conditions)**

|  | **Estimate** | **SE** | **90% CI** | $\hat{\boldsymbol{R}}$ | **Bulk ESS** | **Tail ESS** |
| --- | --- | --- | --- | --- | --- | --- |
| Intercept | -1.21 | 0.61 | -2.18, -0.19 | 1 | 5728 | 7667 |
| Alpha | 0.45 | 0.61 | -0.53, 1.44 | 1 | 9094 | 8837 |
| Session | 0.42 | 0.11 | 0.25, 0.60 | 1 | 8373 | 8672 |
| Interaction effect | 0.01 | 0.11 | -0.16, 0.19 | 1 | 10289 | 9041 |
| brms model statement: Successes \| trials (trials) ~ alpha * session +  (1+session\|participant) + (1\|sentence) + (1\|obs) | | | | | | |

*Notes:* SE = Standard Error. ESS = Effective Sample Size. $\hat{R}$ = Gelman-Rubin potential scale reduction statistic. CI = Credible Interval. CTenv in the alpha band was z-scored and session was centered.


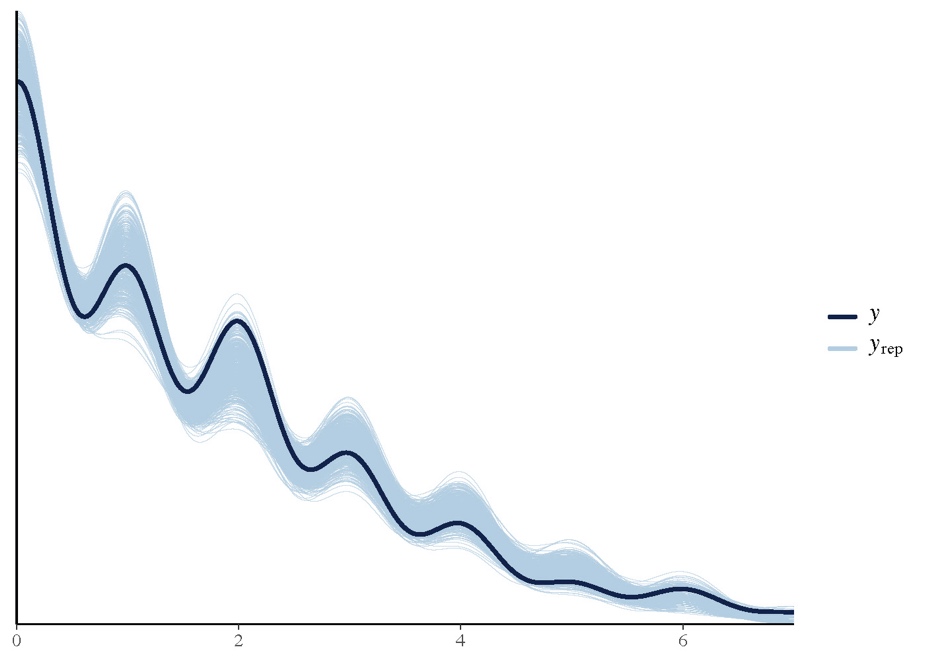


**Figure S7.** Posterior predictive check for the model reported in Table S4. Observed word accuracy is depicted in dark blue and simulated word accuracy is depicted in light blue. The x-axis is the number of correct responses, and the y-axis is the frequency in which the number of correct responses was observed.

**Table S5. Delta (rhythm-enhanced conditions)**

|  | **Estimate** | **SE** | **90% CI** | $\hat{\boldsymbol{R}}$ | **Bulk ESS** | **Tail ESS** |
| --- | --- | --- | --- | --- | --- | --- |
| Intercept | -1.20 | 0.69 | -2.29, -0.10 | 1 | 2965 | 4809 |
| Delta | -0.14 | 0.66 | -1.19, 0.93 | 1 | 3510 | 4241 |
| Session | 0.42 | 0.12 | 0.25, 0.61 | 1 | 4531 | 5978 |
| Interaction effect | 0.01 | 0.12 | -0.17, 0.19 | 1 | 3977 | 4495 |
| brms model statement: Successes \| trials (trials) ~ delta * session +  (1+session\|participant) + (1\|sentence) + (1\|obs) | | | | | | |

*Notes:* SE = Standard Error. ESS = Effective Sample Size. $\hat{R}$ = Gelman-Rubin potential scale reduction statistic. CI = Credible Interval. CTenv in the delta band was z-scored and session was centered.


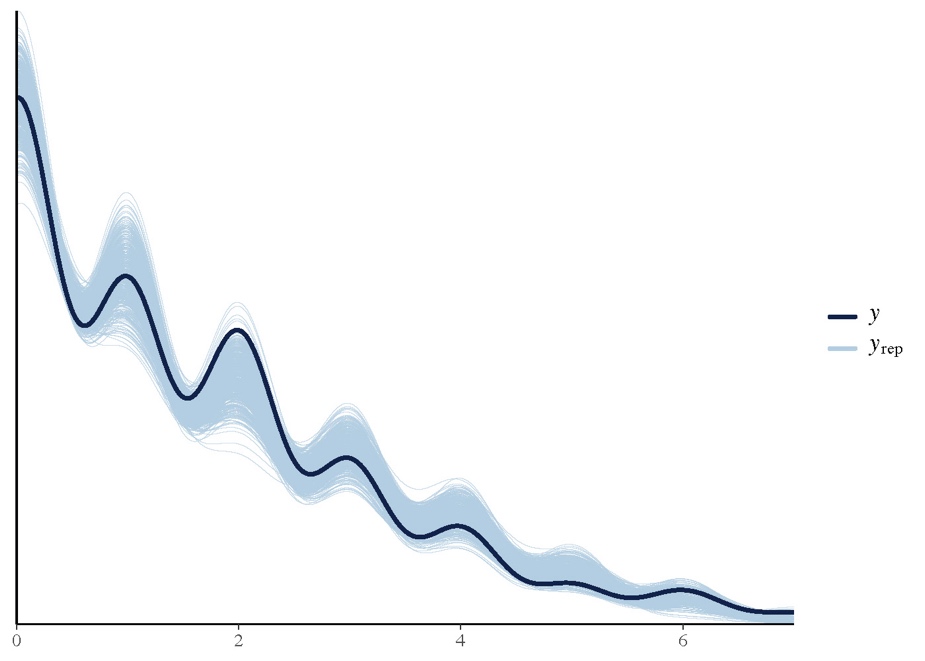


**Figure S8.** Posterior predictive check for the model reported in Table S5. Observed word accuracy is depicted in dark blue and simulated word accuracy is depicted in light blue). The x-axis is the number of correct responses, and the y-axis is the frequency in which the number of correct responses was observed.

**Table S6. Theta (rhythm-enhanced conditions)**

|  | **Estimate** | **SE** | **90% CI** | $\hat{\boldsymbol{R}}$ | **Bulk ESS** | **Tail ESS** |
| --- | --- | --- | --- | --- | --- | --- |
| Intercept | -1.23 | 0.39 | -1.87, -0.59 | 1 | 5273 | 6889 |
| Theta | 1.06 | 0.37 | 0.48, 1.66 | 1 | 6842 | 6972 |
| Session | 0.43 | 0.09 | 0.29, 0.57 | 1 | 8976 | 8894 |
| Interaction effect | -0.12 | 0.09 | -0.27, 0.02 | 1 | 8422 | 8257 |
| brms model statement: Successes \| trials (trials) ~ theta * session +  (1+session\|participant) + (1\|sentence) + (1\|obs) | | | | | | |

*Notes:* SE = Standard Error. ESS = Effective Sample Size. $\hat{R}$ = Gelman-Rubin potential scale reduction statistic. CI = Credible Interval. CTenv in the theta band was z-scored and session was centered.

*
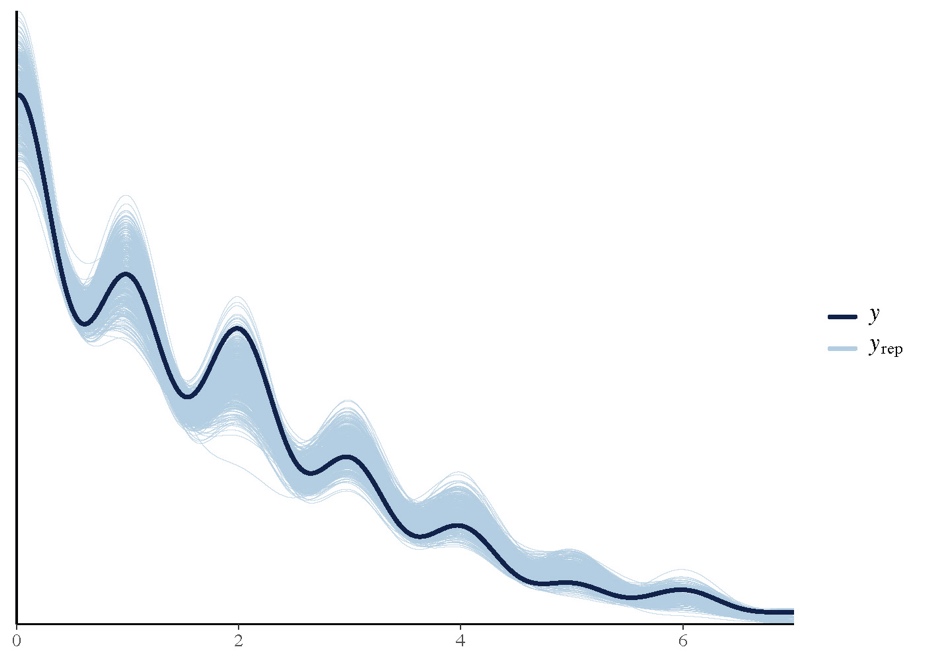
*

**Figure S9.** Posterior predictive check for the model reported in Table S6. Observed word accuracy is depicted in dark blue and simulated word accuracy is depicted in light blue). The x-axis is the number of correct responses, and the y-axis is the frequency in which the number of correct responses was observed.

*
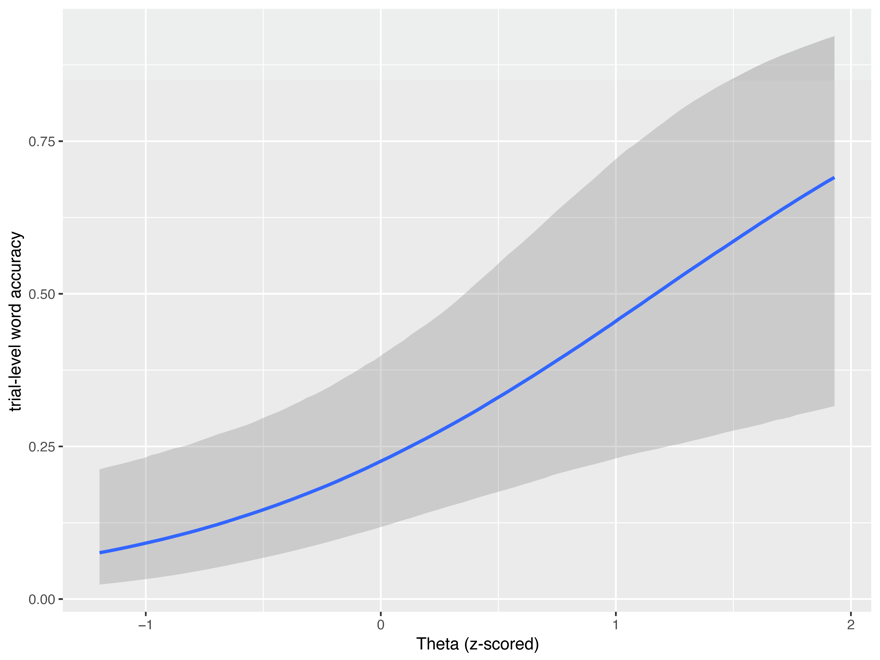
*

**Figure S10.** Main effect of theta for the model reported in Table S6. The x-axis is theta z-scored, and the y-axis is trial-level word accuracy.

**
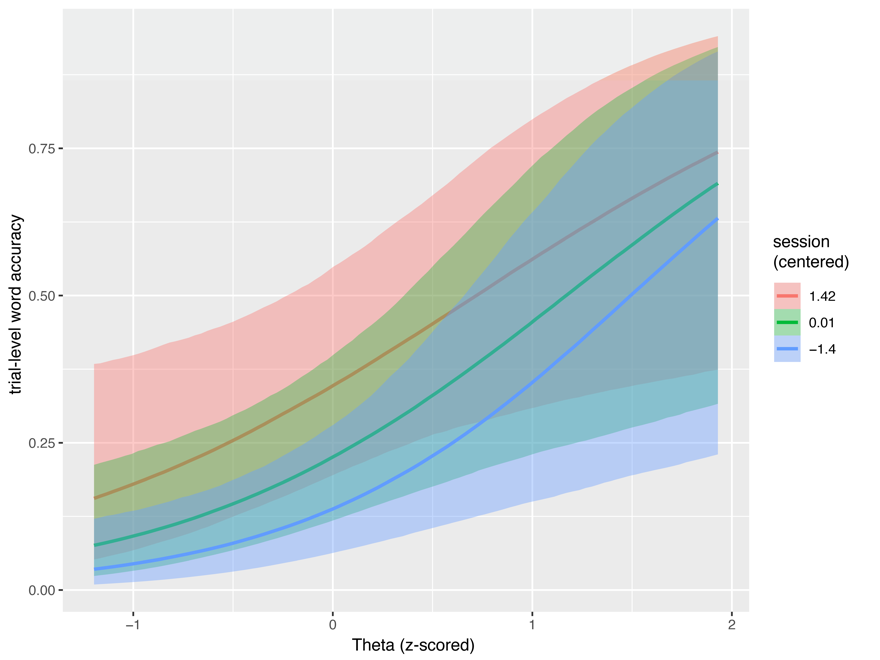
**

**Figure S11.** Interaction effect of theta and session for the model reported in Table S6. The x-axis is theta z-scored, and the y-axis is trial-level word accuracy. Session is centered.

**Table S7. Alpha (control condition)**

|  | **Estimate** | **SE** | **90% CI** | $\hat{\boldsymbol{R}}$ | **Bulk ESS** | **Tail ESS** |
| --- | --- | --- | --- | --- | --- | --- |
| Intercept | -1.27 | 0.61 | -2.26, -0.28 | 1 | 5454 | 7484 |
| Alpha | 0.41 | 0.61 | -0.54, 1.41 | 1 | 8506 | 7819 |
| Session | 0.33 | 0.08 | 0.20, 0.46 | 1 | 10715 | 8852 |
| Interaction effect | 0.00 | 0.08 | -0.13, 0.12 | 1 | 11213 | 8268 |
| brms model statement: Successes \| trials (trials) ~ alpha * session +  (1+session\|participant) + (1\|sentence) + (1\|obs) | | | | | | |

*Notes:* SE = Standard Error. ESS = Effective Sample Size. $\hat{R}$ = Gelman-Rubin potential scale reduction statistic. CI = Credible Interval. CTenv in the alpha band was z-scored and session was centered.

*
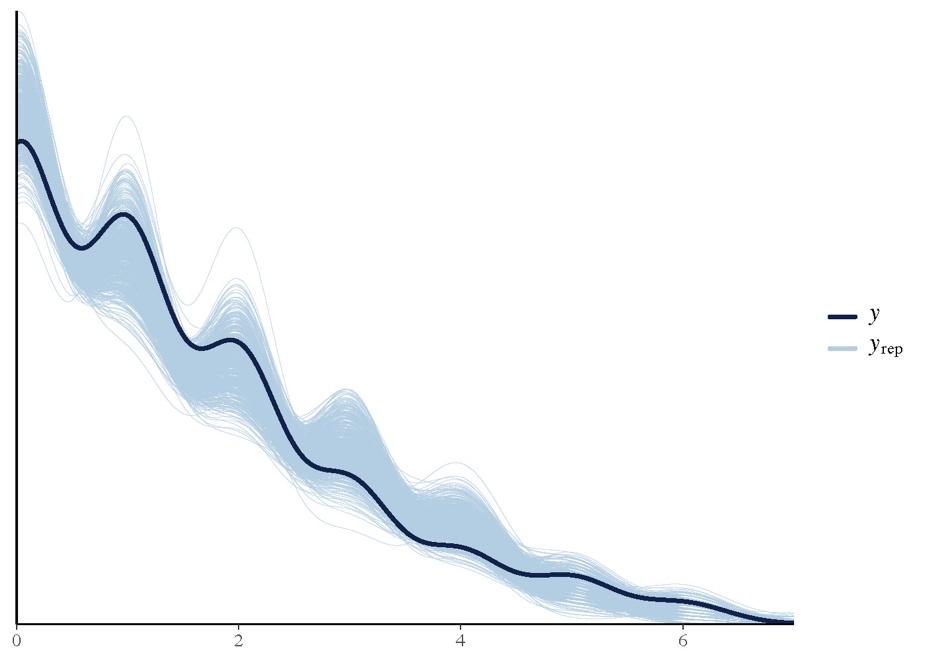
*

**Figure S12.** Posterior predictive check for the model reported in Table S7. Observed word accuracy is depicted in dark blue and simulated word accuracy is depicted in light blue). The x-axis is the number of correct responses, and the y-axis is the frequency in which the number of correct responses was observed.

**Table S8. Delta (control condition)**

|  | **Estimate** | **SE** | **90% CI** | $\hat{\boldsymbol{R}}$ | **Bulk ESS** | **Tail ESS** |
| --- | --- | --- | --- | --- | --- | --- |
| Intercept | -1.28 | 0.61 | -2.24, -0.29 | 1 | 6092 | 7857 |
| Delta | -0.36 | 0.60 | -1.32, 0.58 | 1 | 6897 | 7831 |
| Session | 0.33 | 0.08 | 0.21, 0.46 | 1 | 9883 | 8681 |
| Interaction effect | 0.01 | 0.08 | -0.12, 0.13 | 1 | 10317 | 7510 |
| brms model statement: Successes \| trials (trials) ~ delta * session +  (1+session\|participant) + (1\|sentence) + (1\|obs) | | | | | | |

*Notes:* SE = Standard Error. ESS = Effective Sample Size. $\hat{R}$ = Gelman-Rubin potential scale reduction statistic. CI = Credible Interval. CTenv in the delta band was z-scored and session was centered.

*
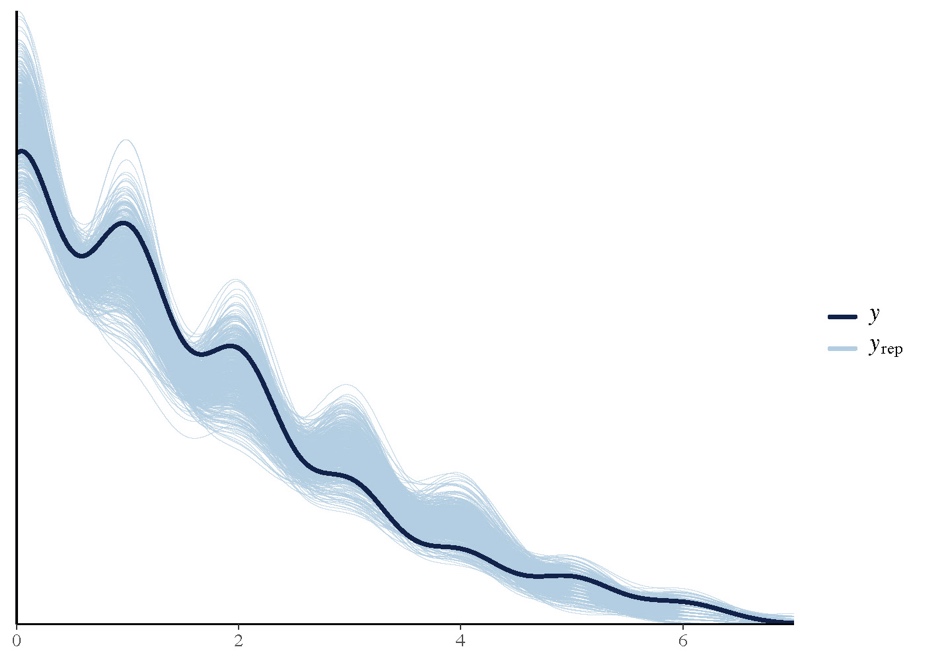
*

**Figure S13.** Posterior predictive check for the model reported in Table S8. Observed word accuracy is depicted in dark blue and simulated word accuracy is depicted in light blue). The x-axis is the number of correct responses, and the y-axis is the frequency in which the number of correct responses was observed.

**Table S9. Theta (control condition)**

|  | **Estimate** | **SE** | **90% CI** | $\hat{\boldsymbol{R}}$ | **Bulk ESS** | **Tail ESS** |
| --- | --- | --- | --- | --- | --- | --- |
| Intercept | -1.30 | 0.51 | -2.1, -0.46 | 1 | 4506 | 6315 |
| Theta | 0.80 | 0.50 | 0.01, 1.59 | 1 | 5397 | 6258 |
| Session | 0.34 | 0.08 | 0.22, 0.46 | 1 | 9434 | 7969 |
| Interaction effect | -0.06 | 0.07 | -0.18, 0.05 | 1 | 9421 | 7768 |
| brms model statement: Successes \| trials (trials) ~ theta * session +  (1+session\|participant) + (1\|sentence) + (1\|obs) | | | | | | |

*Notes:* SE = Standard Error. ESS = Effective Sample Size. $\hat{R}$ = Gelman-Rubin potential scale reduction statistic. CI = Credible Interval. CTenv in the theta band was z-scored and session was centered.


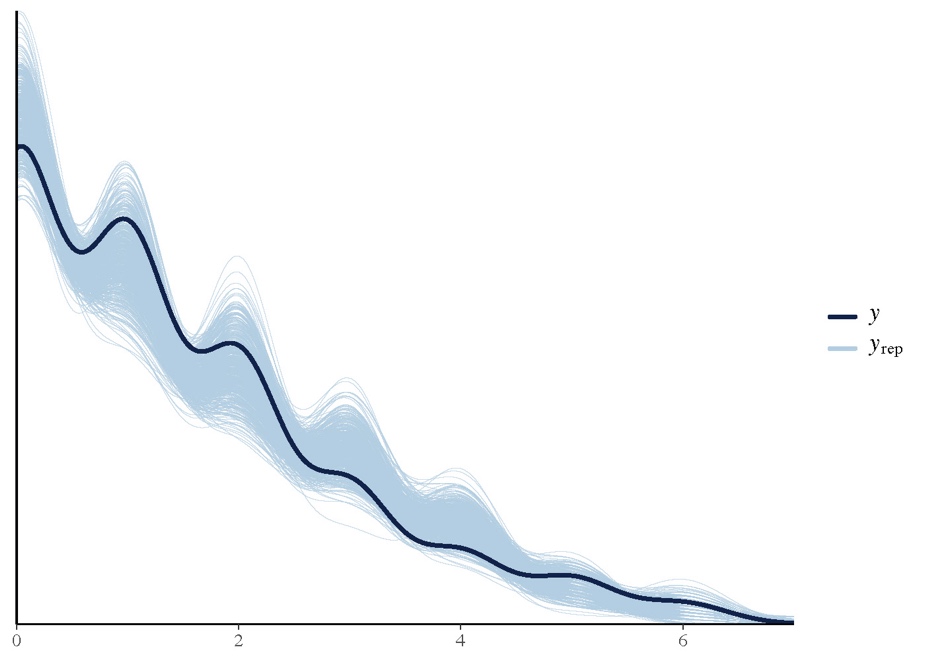


**Figure S14.** Posterior predictive check for the model reported in Table S9. Observed word accuracy is depicted in dark blue and simulated word accuracy is depicted in light blue). The x-axis is the number of correct responses, and the y-axis is the frequency in which the number of correct responses was observed.

**
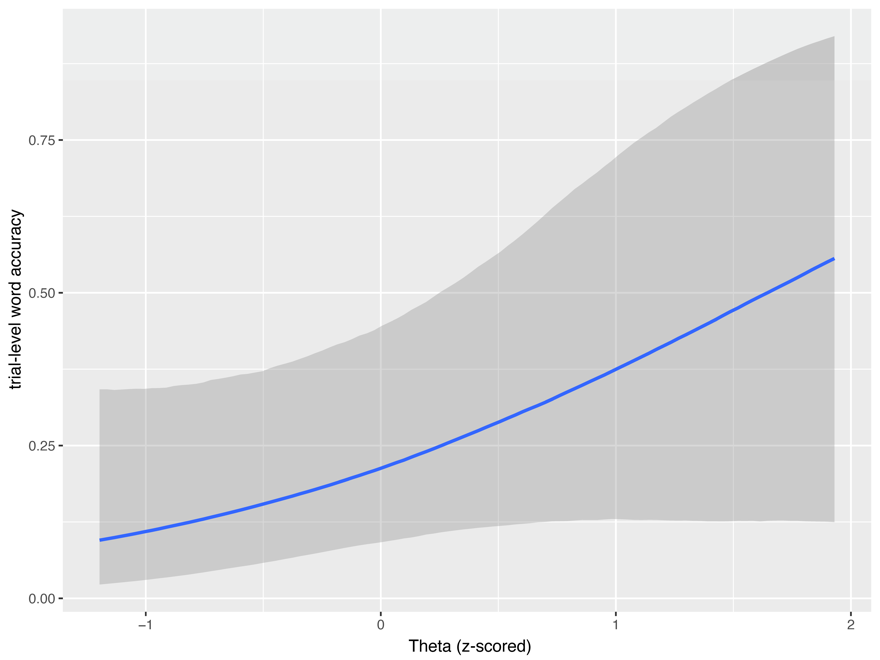
**

**Figure S15.** Main effect of theta for the model reported in Table S9. The x-axis is theta z-scored, and the y-axis is trial-level word accuracy.

**
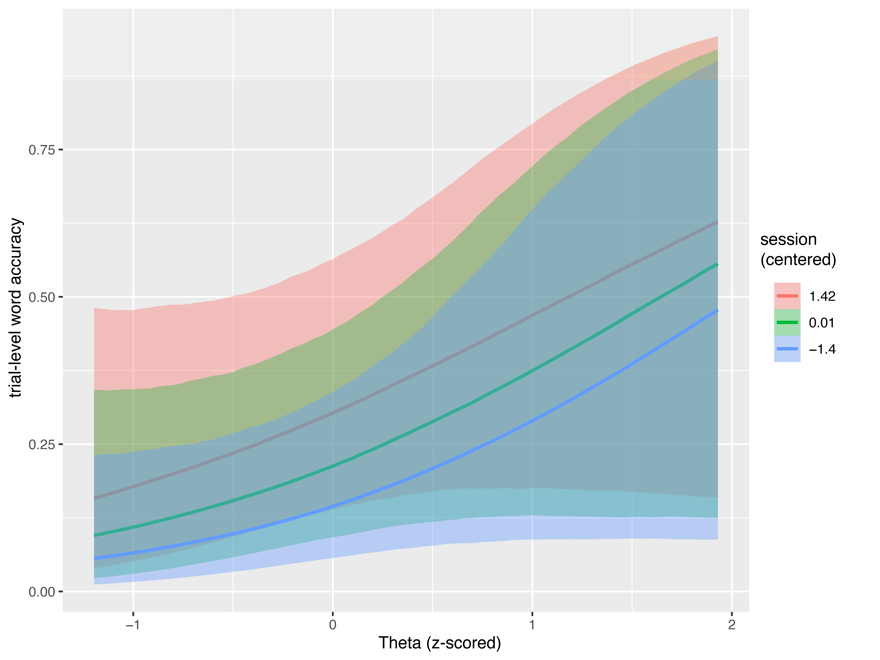
**

**Figure S16.** Interaction effect of theta and session for the model reported in Table S9. The x-axis is theta z-scored, and the y-axis is trial-level word accuracy. Session is centered.
